# Elucidation of the mechanism of amyloid A and transthyretin formation using mass spectrometry-based absolute quantification

**DOI:** 10.1007/s00428-023-03591-w

**Published:** 2023-07-15

**Authors:** Yukako Shintani-Domoto, Koji L. Ode, Seitaro Nomura, Hiroyuki Abe, Hiroki R. Ueda, Takashi Sakatani, Ryuji Ohashi

**Affiliations:** 1 Department of Diagnostic Pathology, Nippon Medical School Hospital, 1‐1‐5, Sendagi, Bunkyo‐ku, Tokyo, 113‐8603 Japan; 2 Department of Systems Pharmacology, Graduate School of Medicine, The University of Tokyo, Tokyo, Japan; 3 Department of Cardiovascular Medicine, The University of Tokyo Hospital, Tokyo, Japan; 4 Department of Pathology, Graduate School of Medicine, The University of Tokyo, Tokyo, Japan; 5 Laboratory for Synthetic Biology, RIKEN Center for Biosystems Dynamics Research, Suita, Osaka Japan; 6 Department of Integrated Diagnostic Pathology, Nippon Medical School, Tokyo, Japan

**Keywords:** Amyloidosis, Autopsy, Proteomics, Heart

## Abstract

Amyloidosis is triggered by the truncation of amyloid precursor proteins, causing organ damages. While previous studies found the truncation of amyloid A (AA) and amyloid transthyretin (ATTR) occurs in C- and N-terminal, respectively, the detailed mechanism of the fibril formation remains unclear. Liquid chromatography mass spectrometry is usually applied for a qualitative purpose, and thus quantification of tryptic peptide residue is difficult. We therefore employed a mass spectrometry-based quantification by isotope-labeled cell-free (MS-QBIC) to analyze the truncation processes in amyloid fibrillogenesis of AA and ATTR using the formalin-fixed paraffin-embedded tissues of autopsy cases. In this study, the process of transthyretin from an 'early fibril state' consisting of full-length ATTR to a 'mature ATTR amyloid fibril' with a truncated low-amyloidogenic segment has been mathematically revealed. The amount of full-length ATTR was nine times higher than in mature fibers. Large cohort studies using MS-QBIC may shed light on the clinical significance of amyloid fibrils.

**Supplementary Information:**

The online version contains supplementary material available at 10.1007/s00428-023-03591-w.

Amyloidosis is caused by amyloid precursor proteins forming amyloid fibrils that are deposited in the extracellular matrix, resulting in organ damage. Currently, the number of identified amyloid fibril proteins is 42, of which 18 proteins appear as systemic amyloidosis [1]. Amyloid A (AA) and amyloid transthyretin (ATTR) are relatively common amyloid subtypes, but the mechanism or the structure of amyloid fibril formation remain unclear.

We previously applied the mass spectrometry-based quantification by isotope-labeled cell-free product (MS-QBIC) to formalin-fixed paraffin-embedded (FFPE) tissues of 30 autopsy cases with amyloidosis, including seven AA cases and nine ATTR cases [2]. This quantification approach, the MS-QBIC method uses absolutely quantified isotope-labeled peptides as the quantification control. (Supplemental Fig. 1) [3, 4]. Therefore, differences in ionization efficiency due to differences in peptide sequence do not, in principle, affect the quantitative value (if the peptide to be measured is quantifiable). Note that quantification values were given within the range where the signal value changes linear to the amount of spiked MS-QBIC peptides[2]. This approach has been feasible and useful in classification and analysis of systemic amyloidosis. Herein, we successfully quantified the truncation processes involved in amyloid fibrillogenesis of AA and ATTR by using MS-QBIC.

Serum amyloid A (SAA) consists of 104 amino acids. Native SAA, which adopts a unique four-helix bundle fold stabilized by its long C-terminal tail, exists as a hexamer. The C-terminus of SAA is presumed to be truncated in AA amyloid fibrils; however, it remains unclear whether the C-terminus is genuinely absent or present at an under-detection level. Using MS-QBIC, amino acids (aa) 26–34 and aa 91–103 of SAA peptides were quantified. Aa 26–34 was detected in all 14 AA samples, whereas the C-terminal aa 91–103 were detected in only six AA samples, illustrating that the average aa 26–34/aa 91–103 ratio was only 4.91% (0.95%–8.24%) among these six samples (Fig. 1A, B). This result indicates that the C-terminus is almost absent or may be present in minimal amounts in amyloid deposits. The length of SAA fragments detected in the amyloid fibrils varies and is dependent on the cases and/or the methods employed, shown by a previous study reporting fragments ranging from aa 2–21 to 2–86 [5]. We recently showed that the distribution of C-terminal tryptic peptides differed from that of N-terminal tryptic peptides in FFPE specimens of AA patients using matrix-assisted laser desorption/ionization imaging mass spectrometry [6]. These results demonstrated that the N-terminus plays a critical role in forming AA amyloid fibrils, consistent with the previous report [5]. A possible mechanism for this fibril formation was the activity of an endogenous protease, but the roles of C-terminal peptides in this process require further investigation.

**Fig. 1:**
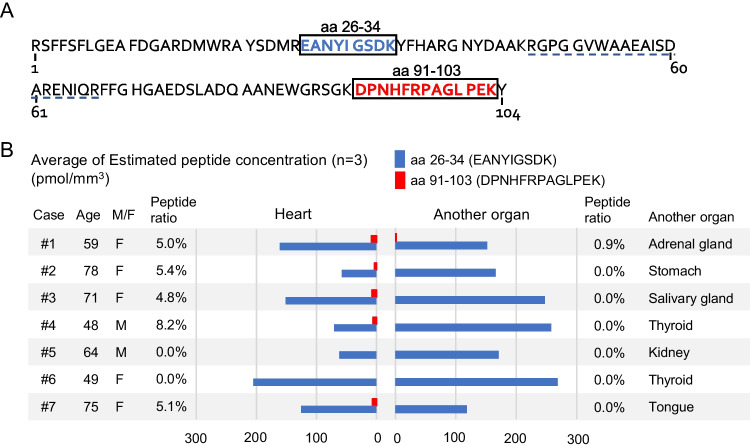
Amino acid sequence and quantitative value of human Serum amyloid A (SAA) peptides. **A**. In our previous study, two peptides, aa 26–34 (blue and bold) and aa 91–103 (red and bold), that are boxed were detected using absolute quantification by liquid chromatography-mass spectrometry (LC–MS) [2]. Residues 47–67 underlined with a blue dotted line are the C-terminal truncation site, which was predominantly detected in a previous report [5]. **B**. Two specimens were taken from each patient: cardiac tissue (cases 1–7, *n* = 7) and another organ (*n* = 7). The bar graph shows the average of three measurements. Residues aa 26–34 were detected in all 14 AA samples, whereas the C-terminal aa 91–103 were detected in only six AA samples, illustrating that the average aa 26–34/aa 91–103 ratio was only 4.91% (0.95%–8.24%) among these six samples

Transthyretin (TTR) consists of 127 amino acids. By using cryo-electron microscopy, Schmidt et al. investigated the misfolding mechanism of TTR, i.e. that aa 36–56 is proteolytically degraded during the process from “early fibril state” to “mature ATTR amyloid fibril” [7]. This early fibril state consists of full-length ATTR and contains the low amyloidogenic segment at aa 36–56 in a solvent-exposed conformation. In our study, we have quantified two tryptic peptides; aa 22–34 and aa 36–48 of ATTR (Fig. 2A, B) that were detected in 47 of 48 samples, with an average aa 36–48/aa 22–34 ratio of 10.26% (2.45%–26.67%), respectively. Quantitative values of aa 36–48 indicated the amount of mature ATTR amyloid fibrils is 9 times larger than the early fibril state (Fig. 2C), supporting the hypothesis of TTR misfolding, proposed by Schmidt et al. [7].

**Fig. 2:**
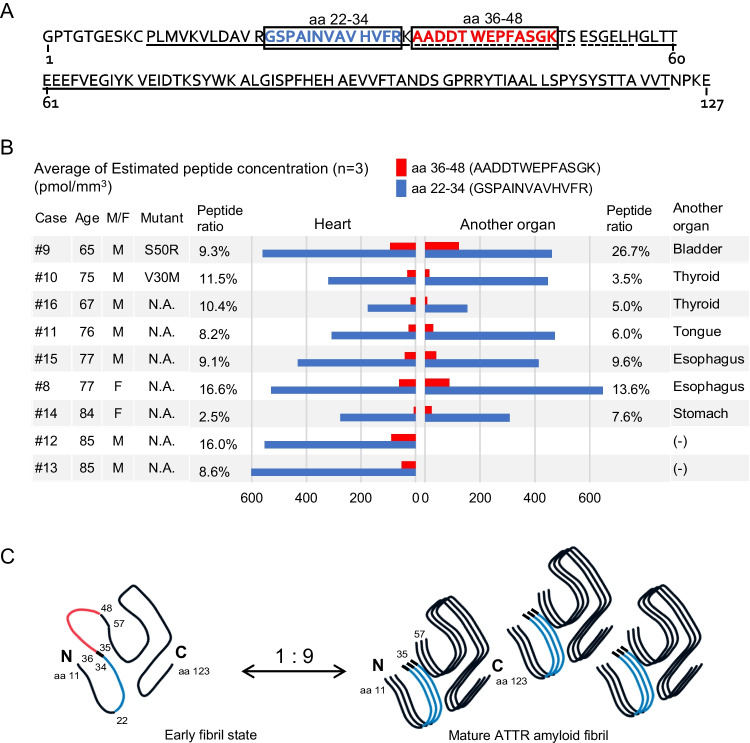
Amino acid sequence, quantitative value of human transthyretin (TTR) peptides and schema of folding TTR based on previously reported data [7]. **A**. Transthyretin (TTR) consists of 127 amino acids. In a previous report using the cryo-EM [7], peptides aa 11–123 were detected (solid underlined) and 35–57 were not (dotted underlined). Two peptides, aa 22–34 (black and bold) and aa 36–48 (red and bold), which were detected using absolute quantification by LC–MS in our previous study [2] are enclosed in the boxes. **B**. We used two specimens from each patient: cardiac tissue (*n* = 9) and another organ (n = 7); however, two ATTR cases were excluded because the amount of deposition in organs other than the heart was too small (cases 15, 16). The bar graph shows the average of three measurements. In our quantification study, two tryptic peptides; aa 22–34 and aa 36–48 of TTR were successfully. Both fragments were detected in 47 of 48 samples, with an average aa 36–48/aa 22–34 ratio of 10.26% (2.45%–26.67%).The original data can be found in “Supporting information (S3 Table)” in reference [2]. **C**. This early fibril state consists of full-length TTR and contains the low amyloidogenic segment at aa 36–56 (including red part). Quantitative values of aa 36–48 indicated mature ATTR amyloid fibrils are 9 times more present than the early fibril state

ATTR has two main fibril morphologies in amyloid fibrils [8]. Type A fibrils are common and consist of N-terminally truncated and full-length TTR. Type B fibrils are formed from only a few mutational variants of TTR and primarily consist of only full-length ATTR. In our study, aa 36–48 is significantly less common than aa 22–34, suggesting that all cases are likely to be ATTR type A. Ihse et al. reported that patients with ATTR fragments (type A) have a late onset of amyloidosis and are more likely to develop cardiomyopathy, while patients without fragments (type B) have an early onset of amyloidosis with a higher incidence of neuropathy and a lower incidence of myocardial involvement [9]. The clinical feature of type A and type B, classified according to the length pattern of amyloid fibrils, is thus markedly different. Large cohort studies using our method may provide new insights into the clinical significance of amyloid fibrils.

Our study using MS-QBIC on amyloid fibrils could mathematically capture the process by which proteolysis converts into mature ATTR amyloid fibrils.

## Supplementary Information

Below is the link to the electronic supplementary material.

Supplementary file 1. Workflow of quantification of amyloid proteins by mass spectrometry–based quantification by isotope-labeled cell-free products (MS-QBIC). A purification tag, a quantification tag, and a tryptic peptide of the target protein (target peptide) are sequentially arrayed as a single peptide sequence (MS-QBIC peptide)[3]. The target peptide sequence is attached by one- or two-step PCR. The MS-QBIC peptide is synthesized in the PURE system in the presence of stable isotope-labeled Arg and Lys for isotopic labeling both the quantification tag and the target peptide [4]. Trypsin digestion of purified MS-QBIC peptide produces equal amounts of isotopically labeled quantification tag and target peptide. The quantification tag is used to measure purified MS-QBIC peptide (left side), and the target peptide is used as an in-ternal standard for the target protein (right side). Modified from Supporting information (S2Fig) in Reference [2]. (PDF 55.8 KB)

## Data Availability

All relevant data are within the paper and its Supporting Information files. The raw data of SRM analysis were deposited to the PeptideAtlas SRM Experiment Library (PASSEL) (https://www.peptideatlas.org/PASS/PASS01558).

## Abbreviations

SAA: Serum amyloid A
AA: Amyloid A
TTR: Transthyretin
ATTR: Amyloid transthyretin
FFPE: Formalin-fixed paraffin-embedded
MS-QBIC: Mass spectrometry-based quantification by isotope-labeled cell-free
LC-MS: Liquid chromatography-mass spectrometry

## References

[1] Buxbaum JN, Dispenzieri A, Eisenberg DS et al (2022) Amyloid nomenclature 2022: update, novel proteins, and recommendations by the International Society of Amyloidosis (ISA) Nomenclature Committee. Amyloid 29:213–219. 10.1080/13506129.2022.2147636 36420821 10.1080/13506129.2022.2147636

[2] Ogawa M, Shintani-Domoto Y, Nagashima Y et al (2020) Mass spectrometry-based absolute quantification of amyloid proteins in pathology tissue specimens: Merits and limitations. PLoS One 15:e0235143. 10.1371/journal.pone.0235143 32609750 10.1371/journal.pone.0235143 PMC7329117

[3] Shimizu Y, Inoue A, Tomari Y et al (2001) Cell-free translation reconstituted with purified components. Nat Biotechnol 19:751–755. 10.1038/90802 11479568 10.1038/90802

[4] Narumi R, Shimizu Y, Ukai-Tadenuma M et al (2016) Mass spectrometry-based absolute quantification reveals rhythmic variation of mouse circadian clock proteins. Proc Natl Acad Sci U S A 113:E3461-3467. 10.1073/pnas.1603799113 27247408 10.1073/pnas.1603799113 PMC4914154

[5] Liberta F, Rennegarbe M, Rösler R et al (2019) Morphological and primary structural consistency of fibrils from different AA patients (common variant). Amyloid 26:164–170. 10.1080/13506129.2019.1628015 31240945 10.1080/13506129.2019.1628015

[6] Shintani-Domoto Y, Sugiura Y, Ogawa M et al (2022) N-terminal peptide fragment constitutes core of amyloid deposition of serum amyloid A: An imaging mass spectrometry study. PLoS One 17:e0275993. 10.1371/journal.pone.0275993 36240260 10.1371/journal.pone.0275993 PMC9565386

[7] Schmidt M, Wiese S, Adak V et al (2019) Cryo-EM structure of a transthyretin-derived amyloid fibril from a patient with hereditary ATTR amyloidosis. Nat Commun 10:5008. 10.1038/s41467-019-13038-z 31676763 10.1038/s41467-019-13038-z PMC6825171

[8] Bergström J, Gustavsson A, Hellman U et al (2005) Amyloid deposits in transthyretin-derived amyloidosis: cleaved transthyretin is associated with distinct amyloid morphology. J Pathol 206:224–232. 10.1002/path.1759 15810051 10.1002/path.1759

[9] Ihse E, Rapezzi C, Merlini G et al (2013) Amyloid fibrils containing fragmented ATTR may be the standard fibril composition in ATTR amyloidosis. Amyloid 20:142–150. 10.3109/13506129.2013.797890 23713495 10.3109/13506129.2013.797890

