# Correction to: Elucidation of the mechanism of amyloid A and transthyretin formation using mass spectrometry-based absolute quantification

**DOI:** 10.1007/s00428-023-03617-3

**Published:** 2023-08-11

**Authors:** Yukako Shintani-Domoto, Koji L. Ode, Seitaro Nomura, Hiroyuki Abe, Hiroki R. Ueda, Takashi Sakatani, Ryuji Ohashi

**Affiliations:** 1 Department of Diagnostic Pathology, Nippon Medical School Hospital, 1‐1‐5, Sendagi, Bunkyo‐ku, Tokyo, 113‐8603 Japan; 2 Department of Systems Pharmacology, Graduate School of Medicine, The University of Tokyo, Tokyo, Japan; 3 Department of Cardiovascular Medicine, The University of Tokyo Hospital, Tokyo, Japan; 4 Department of Pathology, Graduate School of Medicine, The University of Tokyo, Tokyo, Japan; 5 Laboratory for Synthetic Biology, RIKEN Center for Biosystems Dynamics Research, Suita, Osaka Japan; 6 Department of Integrated Diagnostic Pathology, Nippon Medical School, Tokyo, Japan

**Correction to: Virchows Archiv**

https://doi.org/10.1007/s00428-023-03591-w

The image presented in Figure 2 of the published version of the above article was incorrect. The corrected Figure 2 is shown as follows:

**Fig. 2:**
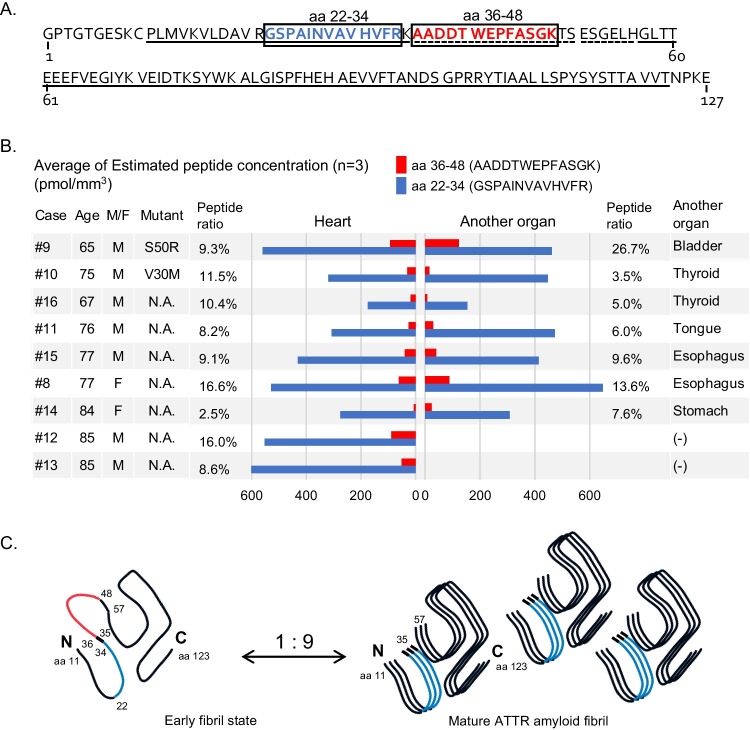
Amino acid sequence, quantitative value of human transthyretin (TTR) peptides and schema of folding TTR based on previously reported data [7]. **A**. Transthyretin (TTR) consists of 127 amino acids. In a previous report using the cryo-EM [7], peptides aa 11–123 were detected (solid underlined) and 35–57 were not (dotted underlined). Two peptides, aa 22–34 (black and bold) and aa 36–48 (red and bold), which were detected using absolute quantification by LC–MS in our previous study [2] are enclosed in the boxes. **B**. We used two specimens from each patient: cardiac tissue (*n* = 9) and another organ (n = 7); however, two ATTR cases were excluded because the amount of deposition in organs other than the heart was too small (cases 15, 16). The bar graph shows the average of three measurements. In our quantification study, two tryptic peptides; aa 22–34 and aa 36–48 of TTR were successfully. Both fragments were detected in 47 of 48 samples, with an average aa 36–48/aa 22–34 ratio of 10.26% (2.45%–26.67%).The original data can be found in “Supporting information (S3 Table)” in reference [2]. **C**. This early fibril state consists of full-length TTR and contains the low amyloidogenic segment at aa 36–56 (including red part). Quantitative values of aa 36–48 indicated mature ATTR amyloid fibrils are 9 times more present than the early fibril state

The original article has been corrected.

