# Supplementary figures and images for: Elucidation of the mechanism of amyloid A and transthyretin formation using mass spectrometry-based absolute quantification
Source: Virchows Arch. 2023 Jul 15;485(5):943–6. doi: 10.1007/s00428-023-03591-w (PMC11564350; doi:10.1007/s00428-023-03591-w)

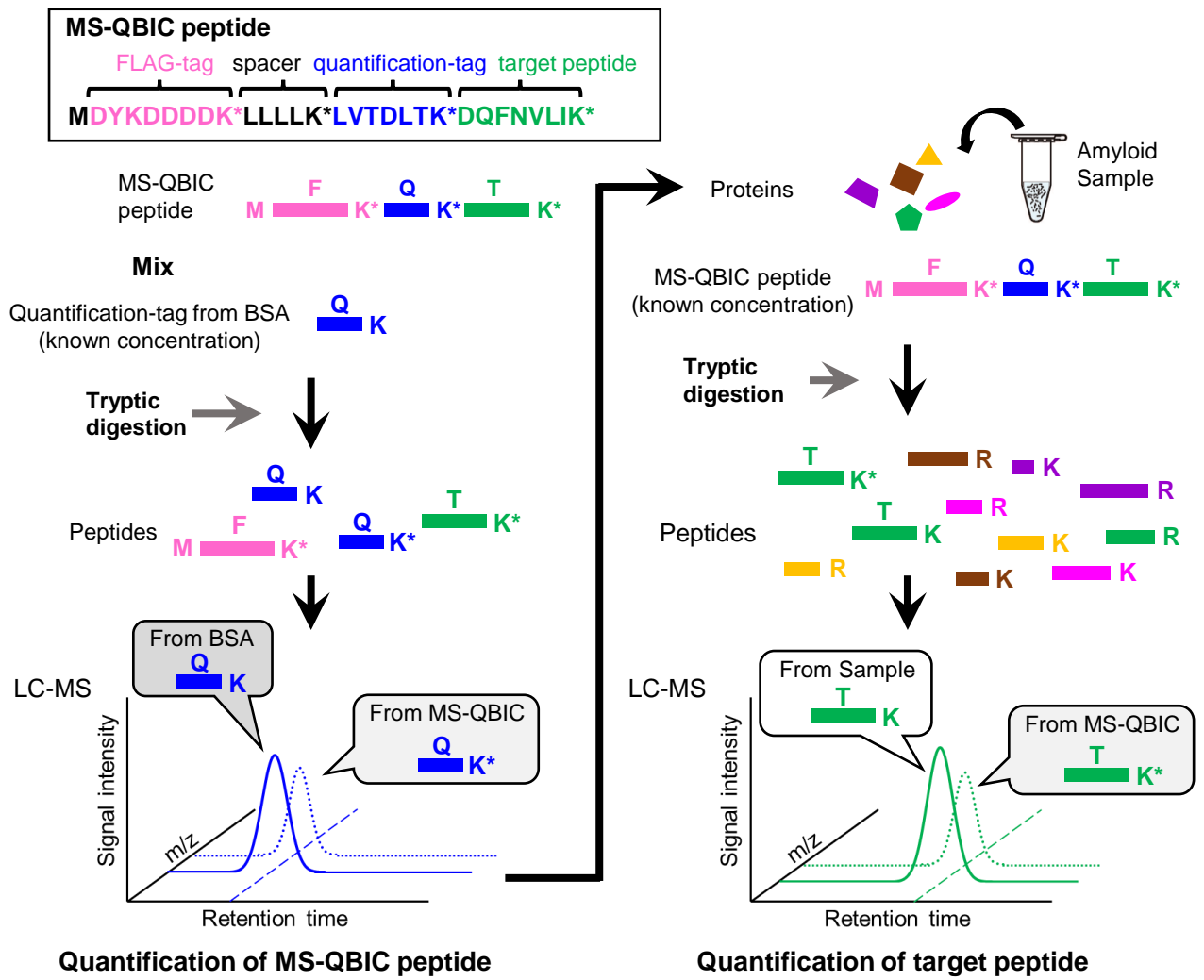

Supplemental Figure 1. Peptide quantification by the MS-QBiC method

Supplement: Supplementary file 1 — Workflow of quantification of amyloid proteins by mass spectrometry–based quantification by isotope-labeled cell-free products (MS-QBIC). A purification tag, a quantification tag, and a tryptic peptide of the target protein (target peptide) are sequentially arrayed as a single peptide sequence (MS-QBIC peptide)[3]. The target peptide sequence is attached by one- or two-step PCR. The MS-QBIC peptide is synthesized in the PURE system in the presence of stable isotope-labeled Arg and Lys for isotopic labeling both the quantification tag and the target peptide [4]. Trypsin digestion of purified MS-QBIC peptide produces equal amounts of isotopically labeled quantification tag and target peptide. The quantification tag is used to measure purified MS-QBIC peptide (left side), and the target peptide is used as an in-ternal standard for the target protein (right side). Modified from Supporting information (S2Fig) in Reference [2]. (PDF 55.8 KB) [file 428_2023_3591_MOESM1_ESM.pdf]
